# Supplementary material for: Insights into the expanding phenotypic spectrum of inherited disorders of biogenic amines
Source: Nat Commun. 2021 Sep 20;12:5529. doi: 10.1038/s41467-021-25515-5 (PMC8452745; doi:10.1038/s41467-021-25515-5)
Supplement: Supplementary file 1 — Supplementary Information [file 41467_2021_25515_MOESM1_ESM.pdf]

Supplementary Information

Index

Supplementary table 1 ____ 2

Supplementary table 1

<table>
<tr><td>Disease</td><td colspan="3">Maternal health problems during term pregnancies</td><td colspan="2">Maternal drug treatment</td></tr>
<tr><td></td><td colspan="3">n</td><td colspan="2"></td></tr>
<tr><td>AADCD</td><td rowspan="4">30</td><td colspan="2" rowspan="4">Frequent: diabetes (n=6), thyroid disease (n=4). Less frequent (n=1-3): hypertension, pre-eclampsia, vaginal bleeding, decreased fetal movements, psychiatric problems, headache, visual impairment, oligo-/polyhydramnios, mild proteinuria, thrombophilia, anemia, infections (mononucleosis, varicella, vaginal candida), impaired glucose tolerance, uterine myoma and ovarian cysts</td><td colspan="2" rowspan="4">Anti-coagulants (acetylsalicylic acid, heparin), anti-inflammatory drugs, hormones (thyroid hormones, progesterone), anti-depressants, iron, vitamins</td></tr>
<tr><td>THD</td></tr>
<tr><td>MAOAD</td></tr>
<tr><td>DATD</td></tr>
<tr><td>arGTPCHD</td><td rowspan="6">27</td><td rowspan="6">Frequent: diabetes (n=8). Less frequent (n=1-2): hypertension, cardiac dysfunction, iron deficiency, gestosis, hyperemesis gravidarium and abdominal pain</td><td>Dystonia (n=1)</td><td rowspan="6">Iron, magnesium, vitamins, hormones (thyroid hormones, progesterone), H2 receptor antagonist, antibiotics</td><td>L-dopa/ carbidopa (n=1)</td></tr>
<tr><td>adGTPCHD</td><td>Segawa syndrome (n=2), Segawa syndrome together with myasthenia gravis and thyrotoxicosis (n=1), pre-eclampsia (n=1), threatened preterm labor (n=1)</td><td>L-dopa and pyridostigmine (n=1)</td></tr>
<tr><td>PTPSD</td><td>Vaginal bleeding, early contractions, suspected preterm delivery, placental insufficiency, pathological CTG (n=1-2 each)</td><td rowspan="4"></td></tr>
<tr><td>DHPRD</td><td rowspan="3"></td></tr>
<tr><td>SRD</td></tr>
<tr><td>DNAJC12D</td></tr>
</table>

Supplementary table 1: Maternal health problems and drug treatment during term pregnancies.
